# Amniotic Allograft Implantation for Midface Aging Correction: A Retrospective Comparative Study with Platelet-Rich Plasma

**DOI:** 10.1007/s00266-019-01422-5

**Published:** 2019-06-25

**Authors:** Alissa Davis, Adam Augenstein

**Affiliations:** Charlotte, NC USA

**Keywords:** Amnion, PRP, Aesthetics, Injectable, Midface, Anti-aging

## Abstract

Amniotic allografts are becoming more popular for use in soft tissue growth in many areas of medicine because of their immunoprivilege that allows them to proliferate into tissues without rejection by the host. Platelet-rich plasma (PRP) has crossed over from wide orthopedic uses to the aesthetic market for hair restoration and midface volume replacement, owing, in part, to the minimal risk associated with the procedure and the convenience of in-office application. In addition, growth factors provided by PRP help stimulate collagen synthesis in the aging face. However, the potential recruitment of the patient’s own mesenchymal stem cells to the PRP injection site would produce the most favorable and sustained aesthetic outcome. With the advancement of amniotic allograft procedures, the introduction of live mesenchymal cells of the amniotic membrane into the aging midface could be performed in-office similarly to the PRP treatment. This retrospective chart review compares aspects of the amniotic allograft procedure (office time, level of comfort, and downtime) with the aesthetic results of injection into the midface of those undergoing PRP therapy. Analysis of the changes to midface volume, specifically the Ogee curve, observed in the chronological progression of photographs illustrates aesthetic improvements in both PRP and amnion allograft treatment groups, with changes in the facial grading scale. Less patient downtime and slightly more rapid improvements were noted in the amnion group in comparison with the PRP treatment participants.

*Level of Evidence IV* This journal requires that authors assign a level of evidence to each article. For a full description of these Evidence-Based Medicine ratings, please refer to the Table of Contents or the online Instructions to Authors www.springer.com/00266.

## Background

### Autologous Versus Allogenic Cells for Potential Anti-aging Benefits

Regenerative medicine has become more popular in aesthetics with the use of autologous cells, derived from adipose cells and platelet-rich plasma, for the potential tissue proliferation from various growth factors and mesenchymal stem cells [1]. Mesenchymal stem cells have the ability to regenerate by direct tissue differentiation. Additional therapeutic properties have been identified in these cells, including immunomodulation [2]. A variety of molecules, such as growth factors, are released from mesenchymal stem cells in response to injury. Mesenchymal stem cells of the amniotic membrane suppress the inflammatory response by releasing anti-inflammatory molecules that suppress specific pro-inflammatory markers such as transforming growth factor beta (TGF-β), which is associated specifically with a fibrotic response leading to scar formation. In addition, a form of hyaluronic acid (HA) called heavy chain HA is uniquely found in amniotic tissues [2].

Amniotic mesenchymal and epithelial cells release growth factors such as epidermal growth factor, keratinocyte growth factor, and hepatocyte growth factor, all involved in epithelialization and wound healing. Additionally, they facilitate cell migration and adhesion to the basement membrane. Amniotic tissues have antimicrobial properties, with molecules such as transferrin that may contribute to decreased infection risk [3]. The extracellular matrix of the amniotic membrane contains laminin, heavy chain HA, and collagen, which are associated with scaffolding in tissue engineering. A minimal immune response is attributed to the lack of human leukocyte antigen-A (HLA-A), human leukocyte antigen-B (HLA-B), and human leukocyte antigen-DR (HLA-DR) and is a unique characteristic of placental membranes [2].

The clinical application of allogenic cells from amniotic tissue has been used in medical practice for over a century, with its first documented use in soft tissue for treatment of burns. The use of amniotic allografts for orthopedic purposes dates back to 1938 [4].

Autologous cells, such as platelet-rich plasma (PRP), are an excellent source for tissue engineering, with a low risk of immune complications. Limitations exist, however, owing to the quality of cells related to the chronological age of the source patient. Furthermore, underlying conditions of the source patient may result in lack of growth factors and inhibit the potential migration of mesenchymal stem cells to the area of the PRP injection. The use of allogenic cells for tissue engineering offers uniformity, standardization of procedure, and quality control when compared to autologous cells [5] used in other medical disciplines.

### Amniotic Versus Embryonic Allografts

Ethical concerns and lack of availability of human embryonic stem cells (HESCs) have taken the focus from using HESCs to amniotic epithelial cells (AECs). Regarding tissue formation, amniotic stem cells compare favorably to embryonic stem cells for tissue regeneration, as reported by the 2007 Ilancheran et al. study [6]. The authors found that clonogenicity, or the ability of a single cell to form a cloned colony and initiate self-renewal, is comparable to that of HESCs. The authors also showed that AECs do not form teratomas when transplanted in mice, whereas previous studies [7] show teratoma formation in immune-deficient mice injected with HESCs [3]. Another advantage of using amniotic epithelial stem cells for tissue regeneration is their ability to proliferate without the need for a second cell type as a feeding layer. When cultured, the Miki et al. study in 2005 showed a feeding layer formed by AECs at the bottom of the culture dish. This characteristic is important for the attachment of tissues, or scaffolding, in tissue engineering. The same study demonstrated another advantage of AECs: a large number of cells obtained from a single amnion, with an average of over 100 million AECs collected [3].

### Advantages of Autologous or Allogenic Cells Versus Other Constructs Used in Aesthetic Medicine

Procedures for volume replacements in the face currently include surgical implants (non-degradable) and injectables, also categorized as dermal filler (degradable). These procedures can provoke a foreign body reaction when implanted, or delayed inflammatory reactions months after implantation. The stimulation of giant cells and macrophages by a foreign body produces cytokines that attract fibroblasts. These can lead to granuloma formation in dermal filler injection sites [8]. Fibroblasts can be activated by TGF-β. As mentioned above, the amniotic membrane down-regulates TGF-β and its expression and modulates wound healing through tissue reconstruction. There is no risk of host rejection with an amnion allograft, as it is immunologically inert [9]. Recent research by Buday and Ozturk, who transplanted amniotic membrane pieces into the soft tissue of rats’ backs, showed the use of the membrane for injection material without foreign body reaction, necrosis, or fibrosis. Furthermore, the use of amniotic membrane for soft tissue filler was concluded in this study as the membrane held its presence, augmenting the tissue into which it was transplanted [10].

The researchers hypothesized that placement of amniotic allograft into the midface should result in improvement in the Ogee curve using a facial grading scale comparable to that of PRP. These improvements would in theory occur in a shorter time frame than PRP and with less downtime for the patient.

## Materials and Methods

### Patients

This retrospective study included a total of eight patients who underwent either PRP treatment or the amnion allograft. They were divided into two groups: four patients undergoing the PRP treatment and four patients undergoing the treatment with the amnion allograft to the midface region.

In orthopedics, the selection of appropriate candidates to undergo PRP treatment impacts the results [11]. Applying the same principle to the aesthetic patient, chronological age and degree of midface volume loss would be the largest determinant of candidacy for either treatment [12]. Patients seeking procedures to improve this volume loss are almost entirely female. An annual review performed by the American Society of Plastic Surgeons showed that 92% of aesthetic services were performed on female patients. Charts reviewed included patients between the ages 42 and 58 years and all female.

Participants refrained from use of nonsteroidal anti-inflammatory agents, aspirin, or steroids for 2 weeks prior to the date of the procedure as recommended. No participant had a history of thrombocytopenia, mast cell activation syndrome, active infection, or carcinoma. Additionally, patients with autoimmune disorders were excluded given the lack of conclusive data regarding the origin of cells involved in microchimerism [13]. All participants were non-smokers. Charts selected for review had the patients refraining from undergoing any cosmetic procedures post-PRP or amnion treatment for 3 months that would alter the dermis or tissue of the midface. SPF protection and minimal sun exposure to reduce damage caused by sunlight were post-procedure instructions for patients. Data was collected and analyzed from the charts kept on file and stored in a database at a private clinic. Solutions IRB have been overseeing the protection of data from the study.

The Harvest SmartPrep System, a part of Harvest Technologies through Terumo BCT, was utilized for PRP collection having shown advantages for platelet concentration and efficiency of capture [14]. For the amniotic allograft, the Organogenesis ReNu-advanced amniotic allograft was chosen as an established FDA-regulated company with a HCTP 361-registered product. The ReNu amniotic allograft includes all growth factors and extracellular matrix components of the amniotic fluid in addition to containing live, cryopreserved mesenchymal cells. Additionally, the ReNu amniotic allograft is frequently described in orthopedic uses, both in surgical and non-surgical office settings, without adverse events [15]. The primary investigator performed all procedures on patients of the charts reviewed to avoid bias of varying techniques. The study was approved by an IRB.

## Procedure

### ReNu-Advanced Amniotic Allograft

The amniotic allograft was delivered on-site to the surgical office and kept in a cryogenic state prior to patient injection. Once removed from ice, the allograft was mixed, 1 mL of injectable saline to 1 mL of allograft, following established protocol for use in orthopedics [16]. The allograft was allowed to thaw for 5–10 min and carefully placed in a 3 mL syringe for injection within 30 min of its removal from ice.

Topical numbing was applied on the medial to lateral region of the patient’s midface to minimize discomfort; however, per Organogenesis' recommendation, no lidocaine was applied to the allograft itself. After 10 min, the numbing agent was removed and the area cleansed with alcohol and chlorhexidine. Using a patent pending technique, the product was injected in the midface region below the dermis for a total of 1 mL per side. The allograft was injected using a 22-gauge needle following Organogenesis’ recommendations [16]. The viscosity of the amnion allograft was slightly higher than that of PRP, although a slow delivery of product is desired for both patient comfort and to minimize disruption of the amniotic allograft. Ice packs were placed for patient comfort after pressure was applied for coagulation of the puncture wound. Post-procedure pictures were taken within 20 min of injections.

### PRP Treatment

The Harvest SmartPrep System (30 mL kits) was used with the established procedure for uniform PRP collection. A total of 30 mL of blood was drawn from the patient’s antecubital area and placed in a chamber for centrifugation. A numbing agent was topically applied to the injection sites and removed after 15 min. Injection sites were thoroughly cleansed. After centrifugation, the platelet-poor plasma was removed, the buffy coat identified, and the PRP drawn into a 10 mL syringe. A sodium bicarbonate and 2% lidocaine mixture (0.1 mL/0.4 mL, respectively) was added to the PRP [17], followed by 0.25 mL of calcium chloride mixed through a female to female connector. The PRP solution was placed in two 3 mL syringes secured by a 27-gauge needle. The four participants yielded between 4 and 5 mL of PRP each. For comparison and uniform assessment of the midface, 1 mL of PRP was injected into the same midface region using the same technique as the amniotic allograph injection. The remaining PRP was placed in other regions requested by the patient, including temples, upper lip, and neck. A massage was performed to the PRP once injected. Post-procedure pictures were taken.

## Results

**Fig. 1:**
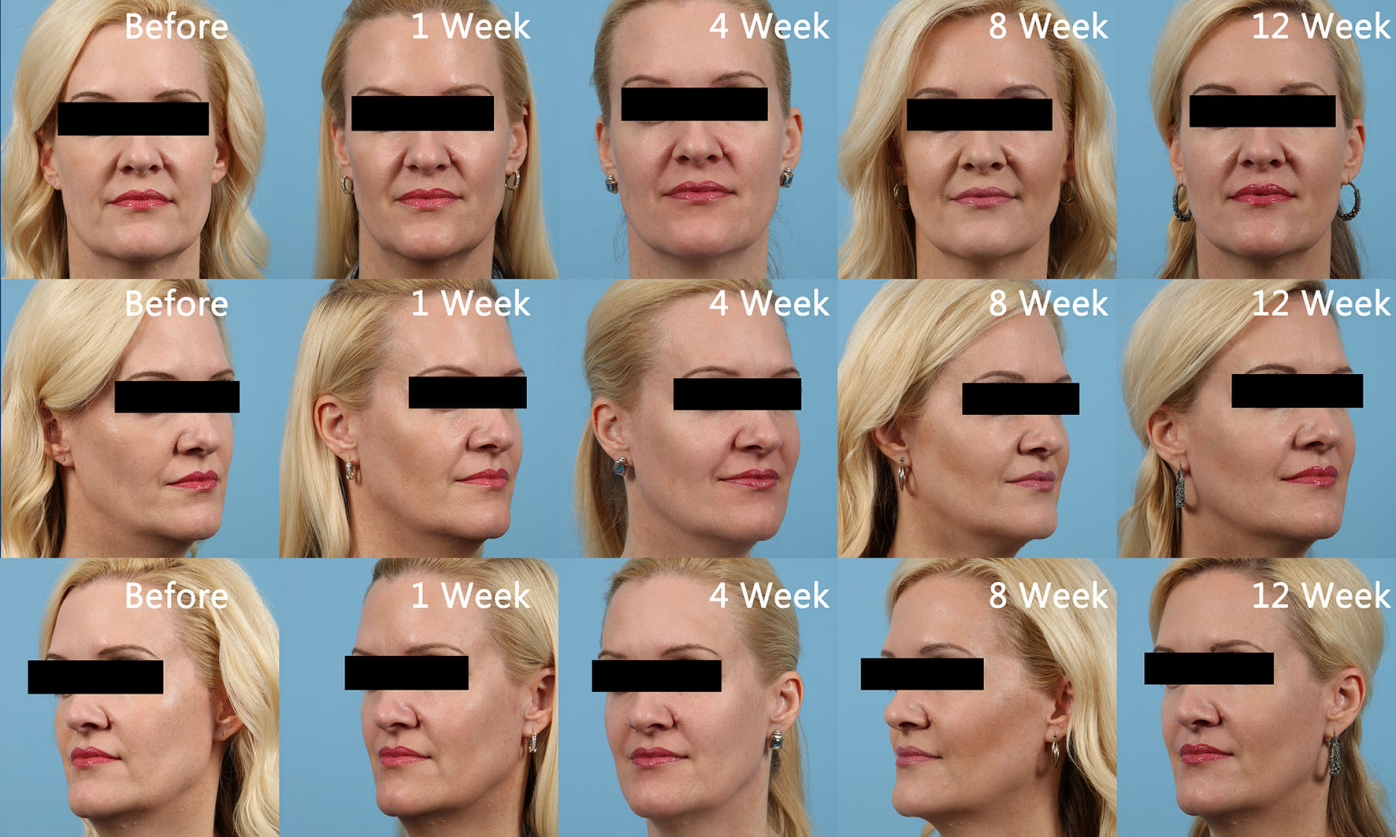
Chronological progression of midface volume loss following treatment with amniotic allograft

**Fig. 2:**
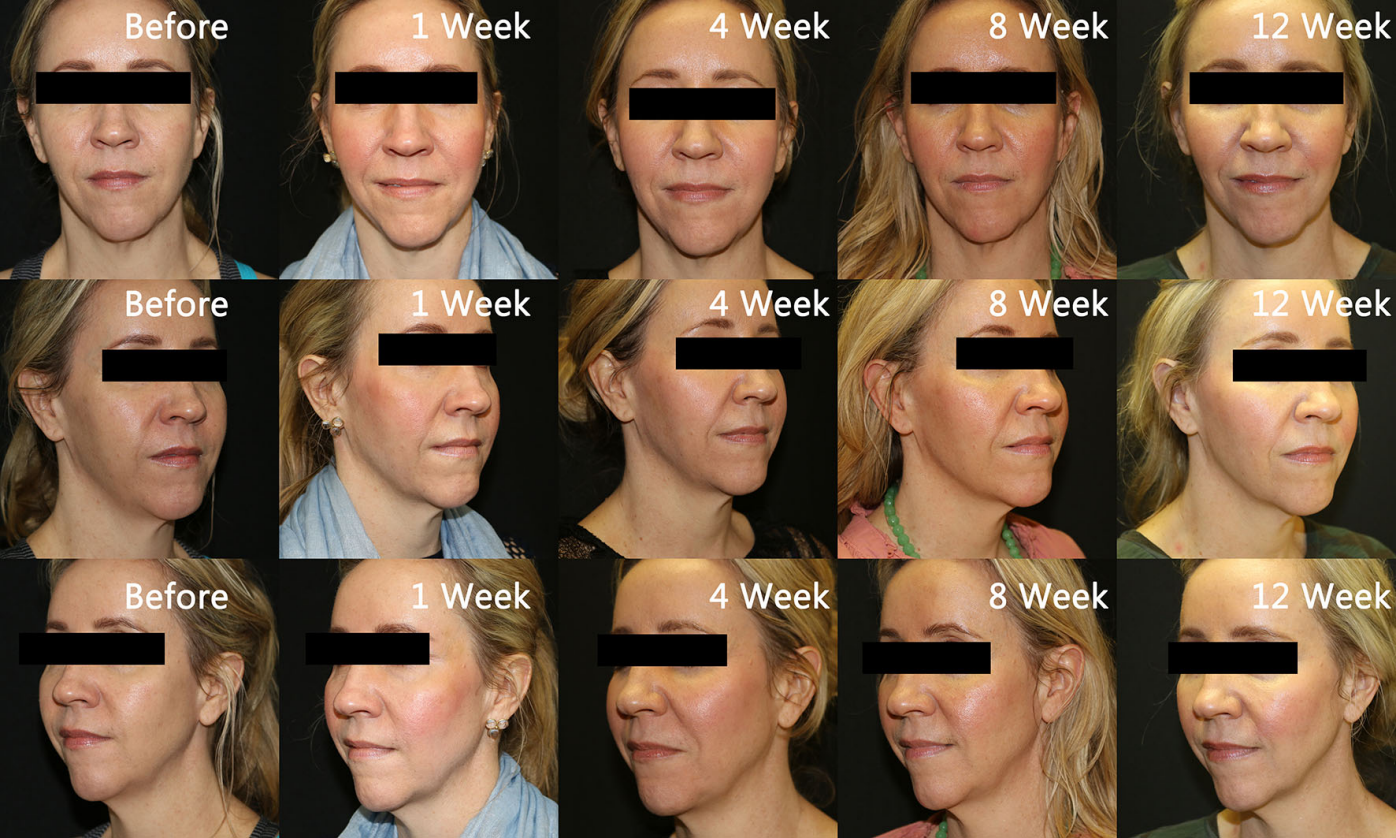
Chronological progression of midface volume loss following treatment with platelet-rich plasma

**Fig. 3:**
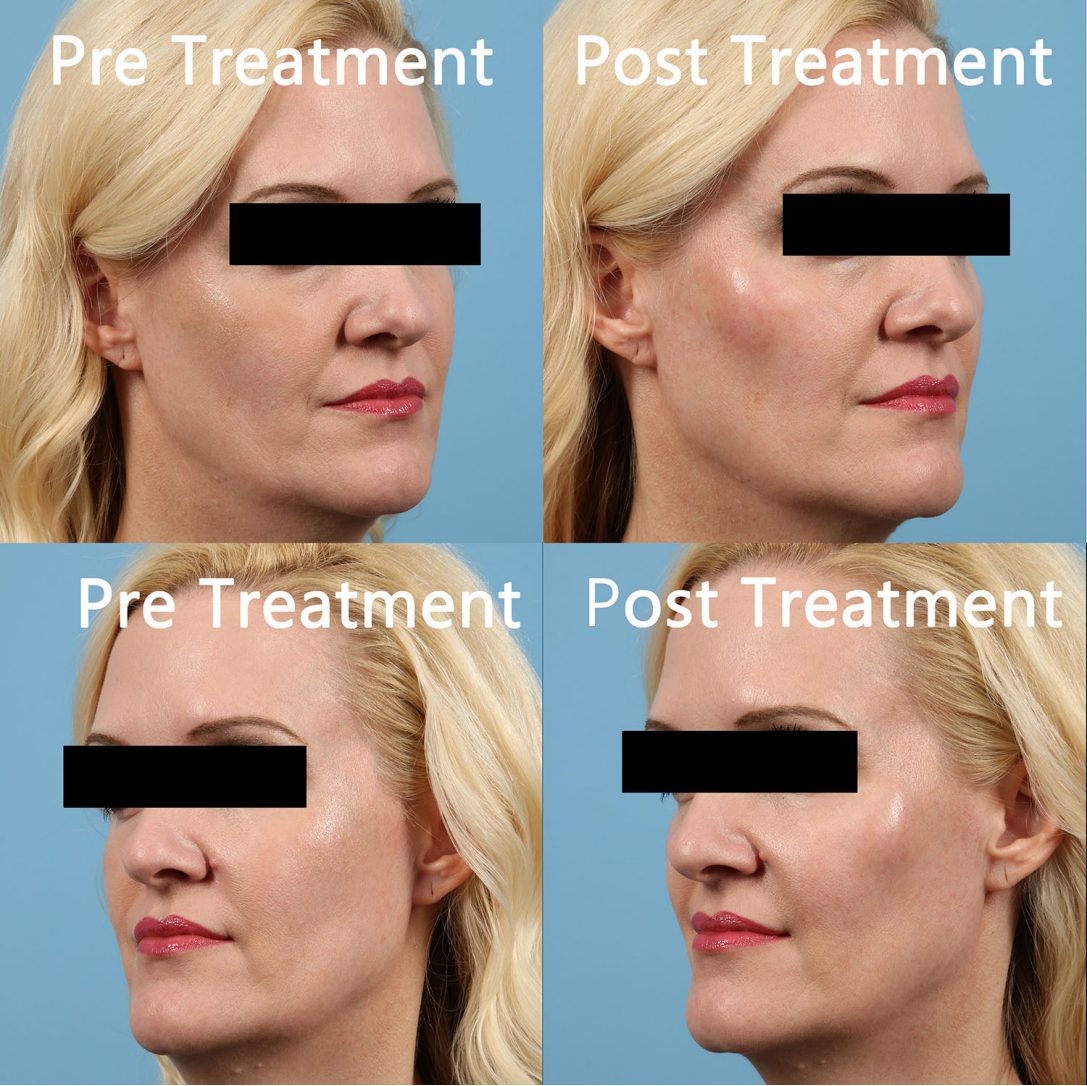
Pre-treatment and immediately post-treatment with amniotic allograft

**Fig. 4:**
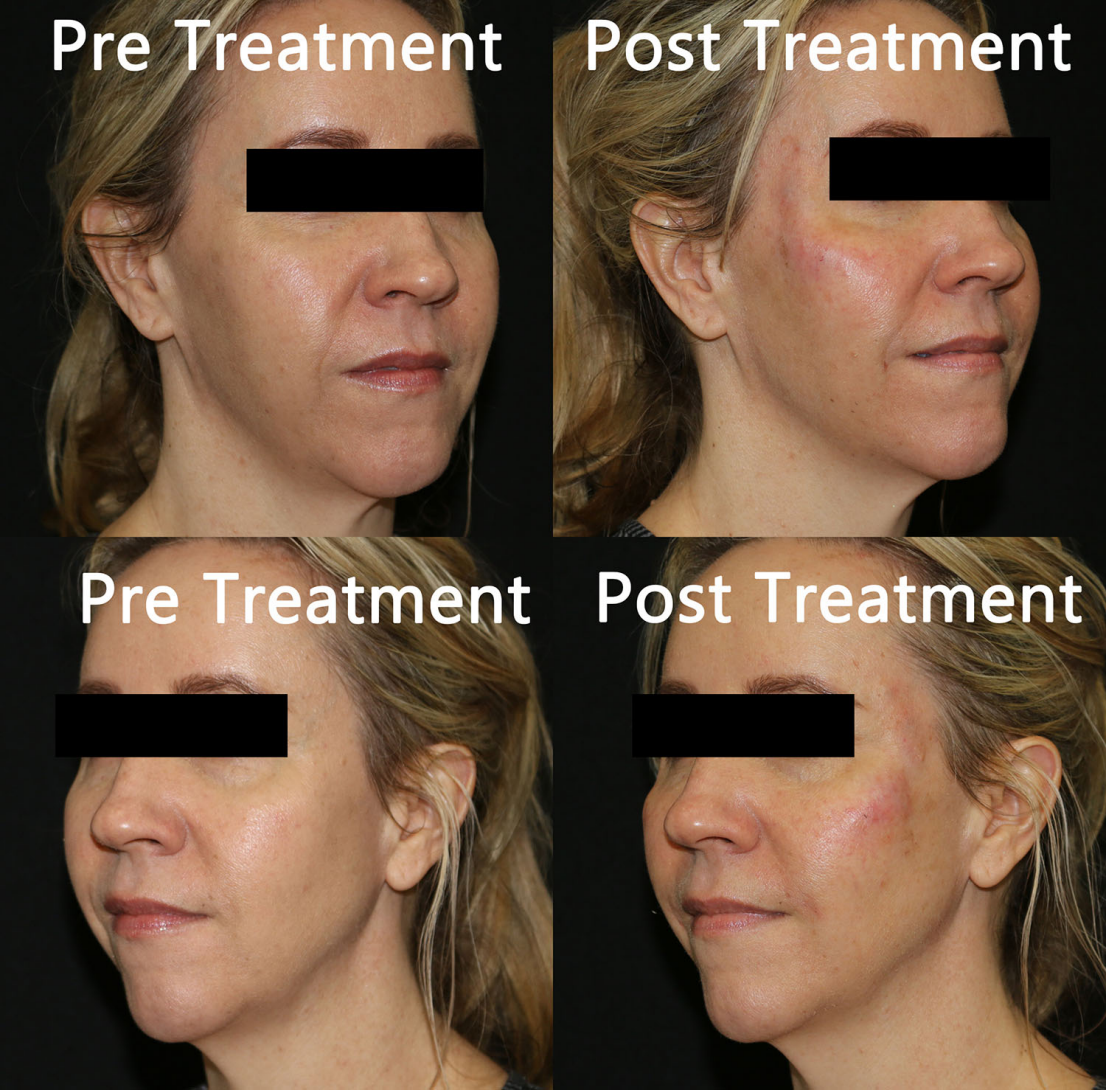
Pre-treatment and immediately post-treatment with platelet-rich plasma

Written records of charts and photographic data placed in chronological order were collected and reviewed by both researchers. Given the nature of aesthetics, it was determined that the effectiveness of the injections would be best assessed by pictures for comparison at baseline, immediately post-procedure, 1 week, 4 weeks, 8 weeks, and 12 weeks post-procedure allowing for maximal achieved results. Pictures show the improvement in tissue volume of the midface, particularly observed in the improvement of the Ogee curve [18], from baseline to post-injection with amnion (Fig. 1). The timing of the changes is also indicated. Figure 2 shows patients treated with PRP. Immediate post-procedure pictures show that the amniotic allograft-injected candidates had little to no change in appearance, except for a mild edema in the injection area (Fig. 3). PRP candidates showed discoloration of injected areas in addition to edema (Fig. 4). Oblique and side views of the study participants allow for clear assessment of the Ogee curve of the midface. The improvement in skin coloration and texture can be observed in frontal views as well as improvement in nasolabial folds, marionette lines, and tear troughs. This can be seen in both procedures, with more pronounced differences for the candidates in their forties. The changes in midface appeared more distinguished with ReNu amniotic allograft versus PRP at 4 weeks post-procedure. Changes in all candidates included some improvement in the Ogee curve score specifically assessed using oblique and side views, with the exception of one PRP participant. Improvements in the Ogee curve score appeared earlier with amniotic allograft than with PRP. Results at 12 weeks post-procedure of both the amniotic allograft and PRP showed improvement in the facial grading scale to the third or fourth degree (a third degree corresponds to improvement in appearance compared to baseline, and a fourth degree corresponds to marked improvement from baseline but not completely optimal). The efficacy of the amniotic allograft results compared to those of PRP at 12 weeks was assessed visually, with the former having a slightly superior result. The researchers are aware that the improvements were subtle and of the subjective nature of visually assessing the photographs. The evaluation of the amnion group as superior could result in part from the visual assessment of the patients in person by the researchers. To quantify results, a facial grading scale was used with an assigned baseline and post-procedure scoring of midface volume loss (Table 1).

**Table 1:** Description of the facial grading scale used in this Table: from oblique angles, the Ogee curve can be assessed by drawing curves along the cheek lines that intercept to assess the degree of the curve and its location as superior or inferior on the face. The facial grading scale was used to determine the degree of volume loss at baseline and the improvement or lack thereof post treatment: 0—full upper cheek, 1—mildly flattened upper cheek, 2—moderately flattened upper cheek, 3—severely flattened upper cheek, 4—very severely flattened upper cheek

Participant (#)	Age (Years)	Treatment received	Initial midface volume loss: facial scale	12 weeks post-treatment volume loss: facial scale
1	43	PRP	2	1
2	47	Amnion	3	2
3	46	PRP	3	2
4	42	Amnion	2	1
5	44	PRP	3	2
6	58	Amnion	4	3
7	58	PRP	3	3
8	49	Amnion	3	2

## Discussion

### Participant Perspective

Comments by patients during and following the procedures were recorded in the written chart and assessed as part of the retrospective review. Specifically, patients were assessed for discomfort levels during and immediately post-procedure, along with side effects from the procedure. Recipients of the amniotic allograft described a burning sensation upon injection that decreased upon completion of the injection (< 3 min) and the immediate application of ice to the site. The pain scale ranged from 2 to 7 on a scale of 1 to 10 per participant report during the injection. One participant described a feeling of tenderness, “like a bruise,” in the cheek. All four participants reported that all discomfort subsided prior to leaving the clinic site within 20 min post-procedure. Next day, follow-up reported two of four participants with small hematomas at the large bore needle injection site, although unilaterally. One participant had a residual edema that was more evident on one side, which subsided 3 days post-injection. No discomfort was reported during follow-up of 1–3 days post-treatment.

Participants receiving PRP treatment reported a mild burning sensation upon injection with a score no greater than 4 on a pain scale of 1–10. Part of the discomfort was reported during massage of the PRP rather than injection itself. Use of lidocaine mixed with PRP, as well as a smaller gauge needle, resulted in less discomfort with the injection when compared to the amniotic allograft injections. Use of lidocaine with the amniotic allograft is not recommended, but no negative impact is described on the effectiveness of PRP when used with anesthesia. All four participants showed residual edema in the midface region on the first day post-procedure. Hematomas were present in three of the four patients injected with PRP, with one recipient experiencing a large hematoma on one side. As PRP contains hematocrit, it is unclear to what extent the hematomas resulted from unabsorbed PRP below the skin versus trauma from the puncture and needle advancement. Tenderness remained with patients for several days. The edema resolved after 3 days in one patient, 4 days in two patients, and the patient showing a large hematoma reported that the edema subsided after 1 week.

### Provider Perspective

The time and preparation of the procedures varied greatly. The amniotic allografts required no blood collection or waiting time in a centrifuge. A period of approximately 10 min was necessary to allow the allograft to thaw after removal from ice and addition of saline to the solution. Packaging and adding multiple labels for tissue logs and documentation was a simple process. The injection time from initial puncture to needle withdrawal was less than 120 s per side. Five to 10 min were spent with each patient post-injection, including applying pressure, ice, and a gentle massage, and conversing with the patient about what they were experiencing. Approximately 30 min was required for the injection in-office with minimal preparation of the injector. In contrast, the PRP procedure required 1 h from start to finish if performed as monotherapy. Use of PRP in aesthetics, however, is generally paired with microneedling or injection of HA filler to obtain optimal results. The preparation was more extensive, though it varied depending on the system used for collection. Overall, more was required in both time and preparation for PRP treatment when compared to the ReNu amniotic allograft injection.

## Conclusion

Our results suggest an aesthetic improvement in midface volume with the amniotic allograft when compared with that of PRP. This is the first report to describe results of a series of human-derived amniotic allograft injections for facial augmentation. The ReNu amniotic allograft was chosen based on no adverse effects reported and on large amounts of data of its use in human soft tissues and joints. Limitations of the study include the small number of patients. Further studies are necessary to establish protocols of amniotic allograft placement concerning both depth and facial region, volume of product required, and longevity of results. Other facial regions that exhibit volume loss from aging can be studied in a similar fashion, as well as the use of amniotic allografts in conjunction with other modalities such as microneedling and simultaneous injection of HA dermal filler.

The ease of in-office procedure, minimal downtime, and expedited results advocate its place in aesthetics. Serving as an alternative for patients that are poor candidates for PRP, the injections with ReNu amniotic allografts will provide an option for patients weary of surgical enhancement and/or dermal filler injections. PRP therapy for aesthetic purposes is used alongside HA-derived fillers. Given that HA is naturally found in amniotic epithelial stem cells, further investigation is warranted for use of amniotic allografts and HA-derived fillers. In theory, the immunoprivileged nature of the amniotic stem cells could decrease the potential of delayed foreign body reaction to dermal fillers when performed in conjunction. Allowing the individual’s genes to guide tissue rejuvenation assures a natural look in anti-aging benefits for both PRP and amniotic allograft injections.

## References

[1] Kim I, Bang SI, Lee SK, Park SY, Kim M, Ha H (2014). Clinical application of allogenic implantation of adipogenic differentiated adipose-derived stem cells. Stem Cells Transl Med.

[2] Friel NA, de Girolamo L, Gomoll AH, Mowry KC, Vines JB, Farr J (2016). Amniotic fluid, cells, and membrane application. Oper Tech Sports Med.

[3] Niknejad H, Peirovi H, Jorjani M, Ahmadiani A, Ghanavi J, Seifalian AM (2008). Properties of the amniotic membrane for the potential use in tissue engineering. Eur Cells Mater.

[4] Shimberg M (1938). The use of amniotic fluid concentrate in orthopaedic conditions. J Bone Joint Surg.

[5] Knight MA, Evans GR (2004). Tissue engineering: progress and challenges. Plast Reconstr Surg.

[6] Ilancheran S, Michalska A, Peh G, Wallace EM, Pera M, Manuelpillai U (2007). Stem cells derived from human fetal membranes display multilineage differentiation potential. Biol Reprod.

[7] Thompson JA, Itskovitz-Eldor J, Shapiro SS, Waknitz MA, Swiergiel JJ, Marshall VS, Jones JM (1998). Embryonic stem cell lines derived from human blastocysts. Science.

[8] Bitterman-Deutsch O, Kogan L, Nasser F (2016). Delayed immune mediated adverse effects to hyaluronic acid fillers: report of five cases and review of the literature. Dermatol Rep.

[9] Anderson JJ, Adeleke AT, Rice B, Swayzee Z (2017). Surgical treatment of peroneus brevis tendon repair with and without human amniotic allograft: a comparison study. Clin Surg.

[10] Buday MC, Ozturk M (2018). Evaluation of folded amniotic membrane and injectable amniotic membrane pieces as soft tissue filler materials. Auris Nasus Larynx.

[11] Foster TE, Puskas BL, Mandelbaum BR, Gerhardt MB, Rodeo SA (2009). Platelet-rich plasma from basic science to clinical applications. Am J Sports Med.

[12] Kaer M, Garg RK, Singla S (2013). Analysis of facial soft tissue changes with aging and their effects of facial morphology: a forensic perspective. Egypt J Forensic Sci.

[13] Di Germanio C, Bernier M, de Cabo R, Barbani B (2016). Amniotic epithelial cells: a new tool to combat aging and age-related diseases?. Front Cell Dev Biol.

[14] Kevy SV, Jacobson MS (2004). Comparison of methods for point of care preparation of autologous platelet gel. J Extra Corpor Technol.

[15] Vines JB, Aliprantis AO, Gomoll AH, Farr J (2015). Cryopreserved amniotic suspension for the treatment of knee osteoarthritis. J Knee Surg.

[16] NuTech Medical (2012) NuCel: product overview, vol 1, pp 1–2

[17] Kevy SV, Jacobson MS (2018) Platelet-rich plasma and local anesthetics. Retrieved from http://orthodoc.aaos.org/williamFBennettMD/PRP_Local%20Anesthetics.pdf. 4 April 2018

[18] Carruthers A, Carruthers J (2010). A validated facial grading scale: the future of facial ageing measurement tools. J Cosmet Laser Ther.

